# Duplications in ADHD patients harbour neurobehavioural genes that are co‐expressed with genes associated with hyperactivity in the mouse

**DOI:** 10.1002/ajmg.b.32285

**Published:** 2015-02-05

**Authors:** Avigail Taylor, Julia Steinberg, Caleb Webber

**Affiliations:** ^1^ MRC Functional Genomics UnitDepartment of PhysiologyAnatomy and GeneticsUniversity of OxfordOxfordUnited Kingdom; ^2^ The Wellcome Trust Centre for Human GeneticsUniversity of OxfordOxfordUnited Kingdom

**Keywords:** copy number variants, CNV, network, pathways, molecular etiology, mouse models

## Abstract

Attention deficit/hyperactivity disorder (ADHD) is a childhood onset disorder, prevalent in 5.3% of children and 1–4% of adults. ADHD is highly heritable, with a burden of large (>500 Kb) copy number variants (CNVs) identified among individuals with ADHD. However, how such CNVs exert their effects is poorly understood. We examined the genes affected by 71 large, rare, and predominantly inherited CNVs identified among 902 individuals with ADHD. We applied both mouse‐knockout functional enrichment analyses, exploiting behavioral phenotypes arising from the determined disruption of 1:1 mouse orthologues, and human brain‐specific spatio‐temporal expression data to uncover molecular pathways common among genes contributing to enriched phenotypes. Twenty‐two percent of genes duplicated in individuals with ADHD that had mouse phenotypic information were associated with *abnormal learning/memory/conditioning* (“*l/m/c*”) phenotypes. Although not observed in a second ADHD‐cohort, we identified a similar enrichment among genes duplicated by eight *de novo* CNVs present in eight individuals with *Hyperactivity* and/or *Short attention span* (“*Hyperactivity/SAS*”, the ontologically‐derived phenotypic components of ADHD). In the brain, genes duplicated in patients with ADHD and *Hyperactivity/SAS* and whose orthologues’ disruption yields *l/m/c* phenotypes in mouse (“candidate‐genes”), were co‐expressed with one another and with genes whose orthologues’ mouse models exhibit hyperactivity. Moreover, genes associated with hyperactivity in the mouse were significantly more co‐expressed with ADHD candidate‐genes than with similarly identified genes from individuals with intellectual disability. Our findings support an etiology for ADHD distinct from intellectual disability, and mechanistically related to genes associated with hyperactivity phenotypes in other mammalian species. © 2015 *The Authors. American Journal of Medical Genetics Part B: Neuropsychiatric Genetics* Published by Wiley Periodicals, Inc.

## INTRODUCTION

Attention deficit/hyperactivity disorder (ADHD) is a common neuropsychiatric disorder with childhood onset, prevalent in approximately 5% of children [Polanczyk et al., 2007] and 1–4% of adults [Kessler et al., 2006; Fayyad et al., 2007]. The personal and societal costs of the disorder are high, including education and employment problems [Pelham et al., 2007; Danckaerts et al., 2010; Adamou et al., 2013], as well as drug and alcohol addiction [Biederman et al., 1995; Schachar and Tannock, 1995; Thapar et al., 2001; Ohlmeier et al., 2008]. ADHD has two subtypes—predominantly inattentive and predominantly hyperactive‐impulsive—which may be present singularly or together in an individual with the disorder (*Diagnostic and Statistical Manual of Mental Disorders* [4th ed., text rev.; *DSM‐IV‐TR*; American Psychiatric Association TR]). In addition, there is significant heterogeneity in the underlying neuropsychological impairments and comorbidities among individuals with ADHD [Spencer et al., 2007; Wahlstedt et al., 2009; Larson et al., 2011].

Family and twin studies have estimated that ADHD has high heritability, ~76% [Faraone et al., 2005], but the genetic etiology of ADHD remains elusive. Recent work suggests that the contribution of common single nucleotide polymorphisms (SNPs) to phenotypic variance is around 25–28% [Cross‐Disorder Group of the Psychiatric Genomics Consortium, 2013], while the additive effects of significantly associated candidate genes contribute only 3.3% to phenotypic variance [Kuntsi et al., 2006]. Furthermore, linkage analyses have confirmed only one associated region on chromosome 16q21–24 [Zhou et al., 2008], and genome‐wide association studies (GWAS) have not provided significant novel associations between any individual SNP and ADHD [Lasky‐Su et al., 2008; Lesch et al., 2008; Neale et al., 2008; Franke et al., 2009; Mick et al., 2010; Neale et al., 2010]. These findings, combined with evidence for a significant polygenic component in the etiology of ADHD [Cross‐Disorder Group of the Psychiatric Genomics Consortium, 2013; Hamshere et al., 2013; Yang et al., 2013], raise the hypothesis that rare variants in many genes may contribute to the disorder. Corroboratively, a significantly increased rate of rare, large (>500 Kb) copy number variants (CNVs) was found in patients with ADHD compared to controls [Williams et al., 2010; Stergiakouli et al., 2012; Williams et al., 2012], although this finding was not replicated in other reports [Elia et al., 2010; Lionel et al., 2011; Jarick et al., 2014]. The contribution of CNVs to the aetiology of ADHD remains poorly understood.

In this study, we explored the hypothesis that distinct CNVs give rise to ADHD by affecting genes participating in shared biological processes, the disruption of which predisposes towards the disorder. We applied mouse‐knockout functional enrichment analyses to genes disrupted by 71 large CNVs (>500 Kb) identified among a meta‐cohort of 902 individuals with ADHD [Elia et al., 2010; Williams et al., 2010; Lionel et al., 2011] and observed a significant enrichment, among copy number gains, of genes whose 1:1 orthologues’ disruption yields an *abnormal learning/memory/conditioning* (“*l/m/c*”) phenotype in mouse. We observed a similar enrichment among eight large *de novo* duplications present in eight individuals described in the DECIPHER database with *Hyperactivity* and/or *Short attention span* (“*Hyperactivity/SAS*”), the ontologically‐derived phenotypic components of ADHD [Firth et al., 2009; Robinson and Mundlos, 2010]. Genes duplicated in patients with ADHD and *Hyperactivity*/*SAS*, and whose orthologues’ disruption yields *l/m/*c in the mouse were significantly co‐expressed in the brain. Furthermore, these genes were significantly co‐expressed with genes whose orthologues’ disruption cause *hyperactivity* phenotypes in the mouse, and were significantly more co‐expressed than similarly identified genes from individuals with intellectual disability, supporting an ADHD‐specific expression association.

## MATERIALS AND METHODS

### CNV Data for Patients With ADHD

We obtained data pertaining to rare, predominantly inherited, CNVs in 902 patients with ADHD (the “ADHD‐meta cohort”) from three studies: 335 cases reported by Elia et al. [Elia et al., 2010] (“Elia cohort”), 319 cases reported by Williams et al. [Williams et al., 2010] (“Williams cohort”), and 248 cases reported by Lionel et al. [Lionel et al., 2011] (“Lionel cohort”) (Supplemental Table SI). Where necessary, CNV coordinates were lifted to Build 36 using liftOver [Hinrichs et al., 2006]. We restricted our analysis to 71 CNVs > 500 Kb contributed by 67 individuals (Table IA). During this study, three further data sets were published of rare CNVs in an additional 1,842 patients not included in the ADHD‐meta cohort [Jarick et al., 2014; Stergiakouli et al., 2012; Williams et al., 2012] (Supplemental Table SI); we combined them into an “ADHD‐replication cohort” consisting of 187 CNVs > 500 Kb, from up to 180 individuals (see Table IB).

**Table I:** Distribution of CNVs Among Individuals in ADHD and Control Cohorts

Cohort	CNVs	CNVs > 500 Kb	Gains	Gains > 500 Kb	Losses	Losses > 500 Kb	Individuals contributing CNVs > 500 Kb
(A) ADHD‐meta cohort							
Elia cohort	222	14	64	10	158	4	14
Williams cohort	40	40	30	30	10	10	37
Lionel cohort	306	17	149	12	157	5	16
Total	568	71	243	52	325	19	67
(B) ADHD‐replication cohort							
Stergiakouli cohort	47	47	35	35	12	12	44
Williams (2) cohort	460	89	299	67	161	22	89^a^
Jarick cohort	51	51	34	34	17	17	47
Total	558	187	368	136	190	51	180^a^
(C) Control cohort							
Shaikh cohort	24478		3327		21151		2026

(A) CNVs arising in the genomes of individuals from three cohorts of patients with ADHD. The three cohorts together comprise the “ADHD‐meta cohort” and were published by Elia et al [Elia et al., 2010] (“Elia cohort”), Williams et al. [Williams et al., 2010] (“Williams cohort”), and Lionel et al. [Lionel et al., 2011] (“Lionel cohort”). The total number of CNVs and large CNVs (>500 Kb) are given, and then separate counts are shown for gains and losses. The last column shows the total number of individuals whose genomes harbor large CNVs. (B) CNVs arising in the genomes of individuals from three additional cohorts of patients with ADHD. These cohorts comprise the “ADHD‐replication cohort” and were published by Stergiakouli et al. [Stergiakouli et al., 2012] (“Stergiakouli cohort”), Williams et al. [Williams et al., 2012], (“Williams (2) cohort”), and Jarick et al. [Jarick et al., 2014] (“Jarick cohort”). (C) Common CNVs arising in the genomes of individuals who are members of an apparently healthy control cohort, published by Shaikh et al. [Shaikh et al., 2009]. CNVs from this control cohort were not filtered by length, so we do not show corresponding totals for CNVs > 500 Kb; similarly the last column shows the number of individuals harboring a CNV of any length.

^a^ The number of individuals contributing CNVs was not published in [Williams et al., 2012]; instead we show the maximum possible number of contributing individuals.

### Assigning Genes to CNVs

Human genes were assigned to CNVs using the Ensembl Ensmart54 database. Supplemental Figures S1A and S1B describe the protocols used to determine which genes were affected by losses (“loss‐genes”) or gains (“gain‐genes”), respectively. Briefly, for loss‐genes we required the disruption of a coding exon in all transcripts of that gene, whereas we required gain‐genes to be completely overlapped by a CNV (Table II). This protocol is demonstrably less prone to length biases associated with genes specifically expressed in the brain and ensures that the protein product of a gene is affected by a CNV whichever transcript is expressed [Webber, 2011; Noh et al., 2013].

**Table II:** CNV‐Genes Annotated With Mouse Phenotypes in ADHD Cohorts

Cohort	Gain‐genes	Gain‐genes minus control gain‐genes	Gain‐genes annotated with mouse phenotypes	Loss‐genes	Loss‐genes minus control loss‐genes	Loss‐genes annotated with mouse phenotypes
(A) ADHD‐meta cohort						
Elia cohort	57	53	4	17	17	4
Williams cohort	130	116	37	192	183	43
Lionel cohort	82	78	18	16	16	5
Total	264	244	58	213	204	48
(B) ADHD‐replication cohort						
Stergiakouli cohort	206	177	51	102	98	26
Williams (2) cohort	406	365	101	227	203	49
Jarick cohort	200	168	46	102	95	28
Total	594	537	144	385	350	92

(A) Numbers of CNV‐genes in the ADHD‐meta cohort and constituent cohorts. Separate totals are shown for gain‐ and loss‐genes. We show the total number of CNV‐genes, the total after control CNV‐genes are filtered out, and of the remaining CNV‐genes we give the number of genes whose 1:1 mouse orthologues are annotated with phenotypes from the Mammalian Phenotype Ontology (MPO) within the Mouse Genome Informatics (MGI) database (termed “genes annotated with mouse phenotypes”). (B) Numbers of CNV‐genes in the ADHD‐replication cohort and constituent cohorts. Columns are labeled as in (A).

### Removing Genes Overlapped by Common CNVs in Apparently Healthy Individuals

We discarded CNV‐genes present in individuals with ADHD if they were also copy changed in the same direction by any of 24,478 common CNVs present in an apparently healthy control cohort of 2,026 individuals [Shaikh et al., 2009] (Table I), because variants affecting these genes are less likely to be highly penetrant. We identified these control CNV‐genes as above (Supplemental Figure S1), although control CNVs of all lengths were included (Table II).

### Mouse Genome Informatics (MGI) Phenotypes

Phenotypes exhibited during mouse gene model experiments are described using the Mammalian Phenotype Ontology (MPO; [Smith and Eppig, 2009]) and recorded in the MGI resource (http://www.informatics.jax.org [Eppig et al., 2007]; downloaded 16/12/11). Using 1:1 human:mouse gene orthology relationships defined by the MGI, we found 6,350 human genes whose orthologues’ disruption yields a recorded phenotype in mouse. The numbers of CNV‐genes annotated with mouse phenotypes in this manner are shown in Table II. When testing for the enrichment of MGI phenotypes among the mouse orthologues of our CNV‐genes, we focused on 158 phenotypes in the MPO's *Behavioural/neurological phenotype* category (see Supplemental Information and Supplemental Figure S2). For each phenotype, we compared the proportion of CNV‐genes whose orthologues’ yielded that phenotype in mouse with the proportion of all genes, annotated with a mouse phenotype, for which the same was true. *P*‐values were obtained by applying the hypergeometric test subject to a false discovery rate (FDR) of <5% [Storey, 2002], and gene length biases checked (see Supplemental Information).

### CNV Data for Patients With *Hyperactivity* and/or *Short Attention Span*

Using DECIPHER ([Firth et al., 2009], see Supplemental Information), in which clinical phenotypes are described using the London Dysmorphology Database (LDD, [Fryns and de Ravel, 2002]), we obtained CNV data for 22 patients with *Hyperactivity* and/or *Short attention span* (“*Hyperactivity/SAS”*; the “Hyper/SAS cohort”). Within the LDD there is no single term that directly describes ADHD. However, using an LDD‐to‐Human Phenotype Ontology (HPO; [Robinson and Mundlos, 2010]) mapping (Supplemental Figure S3), we can ontologically ascribe meaning to LDD terms and relate them to the HPO phenotype of *Attention deficit hyperactivity disorder*. Thus, in DECIPHER a patient with ADHD must be described with one or both of the LDD terms *Hyperactivity* or *SAS*; other keywords may be relevant to ADHD, but there is no ontological requirement for them to be used to describe ADHD. A limitation of the LDD‐to‐HPO mapping is that the relationship between the LDD terms *Hyperactivity/SAS* and the HPO term for ADHD is not symmetric; so a patient described with the LDD terms *Hyperactivity/SAS* does not necessarily have ADHD. Nonetheless, given the heterogeneity in ADHD phenotypes, identifying genes that influence the constituent phenotypes of a disorder is a classical approach to dissecting the genetic basis of complex disease.

In line with the selection criteria for the studies providing our ADHD‐meta cohort [Elia et al., 2010; Williams et al., 2010; Lionel et al., 2011], we selected the Hyper/SAS cohort so that no individual had autism or seizures. However, contrary to the selection criteria used in the ADHD studies, we were unable to exclude intellectual disability (ID; see Supplemental Information), and 21/22 cohort‐members also had ID. We directly address this issue using a human brain‐specific co‐expression network described below (see Supplemental Information). An additional 100 phenotypes were present among the cohort, but these were unlikely to introduce generalized genetic enrichments unrelated to the phenotypes of interest because each additional phenotype was possessed by only a minor fraction of the cohort (see Supplemental Information and Supplemental Figure S4). Therefore, we proceeded without further phenotype‐based exclusions. We required that CNVs were *de novo* and >500 Kb, yielding 8 gains and 13 losses in 20 individuals (Supplemental Table SII). The gains arose in eight individuals: three with *Hyperactivity*, three with *Short attention span* (*SAS*), and two with both phenotypes. We noted that gains >500 Kb present in the Hyper/SAS cohort are longer than those arising in the ADHD‐meta cohort (Supplemental Figure S5; *P* = 0.003 [Wilcoxon rank sum test]) and our statistical approach accounts for variability in CNV length. As above, we identified 166 gain‐genes not present in control‐cohort gain‐genes, of which 55 (33%) were annotated with a mouse phenotype in the MGI.

### BrainSpan Gene Expression Network

We obtained normalized gene expression data from BrainSpan [Allen Institute for Brain Science] based on RNASeq of up to 16 brain regions (see Supplemental Information) from 41 individuals aged from 8 weeks post‐conception to 40 years. Only genes with an RPKM ≥ 1 in ≥5% of the samples were included. A network was built using R‐package WCGNA following the procedure (including parameterization) recommended by the authors [Langfelder and Horvath, 2008], where genes form nodes and the edges between two genes are weighted with their expression correlation coefficient *r*. Conservatively, we used only the sub‐network comprising edges with weight *r *≥ 0.7, corresponding to the strongest 5% of edges (5,679,999 edges, 13,953 genes). We checked our results when we relaxed the threshold on *r* (see Supplemental Information).

### Calculating Empirical P‐Values for the Connectivity of Genes in the Brain Co‐Expression Network

**Figure 1:**
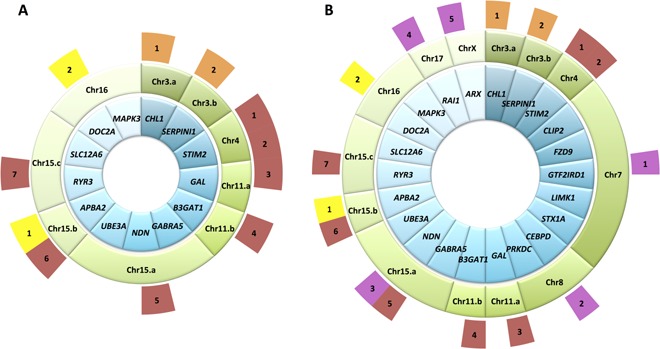
Gain‐genes whose 1:1 mouse orthologues’ disruption yields *abnormal learning/memory/conditioning* in mouse. **A**: Thirteen gain‐genes in patients of the ADHD‐meta cohort had mouse orthologues associated with *l/m/c*. The genes are shown in the innermost, blue, circle. Genes are grouped according to the gains that overlap them as depicted in the middle, green, circle. The outermost circle shows which patients were affected by which gain, and hence which genes were affected in each individual. Patients are colored by cohort: orange = Elia cohort, red = Williams cohort, and yellow = Lionel cohort. **B**: Twenty‐two gain‐genes whose orthologues’ disruption yields *l/m/c*. This is an expanded set comprising the 13 genes shown in Figure 1A, and adding 12 gain‐genes from the Hyper/SAS cohort (three of which are already present in the original set of genes). The concentric circles provide information as described in Figure 1A, but the outermost circle now also shows patients from the Hyper/SAS cohort; these individuals are depicted in bright pink.

Twenty‐two genes contributed to enrichments, observed among gain‐genes in the ADHD‐meta and Hyper/SAS cohorts, of genes whose orthologues’ disruption yields *abnormal learning/memory/conditioning* (*l/m/c*) phenotypes in mouse (Fig. 1). We refer to these genes as “candidate‐genes”. We tested the significance of the connectivity observed among our 22 candidate‐genes, within the brain co‐expression network, by calculating an empirical *P*‐value (*P_emp_*): For 100,000 permutations, we randomly picked 22 genes from the 439 genes whose orthologues when disrupted in mouse yield *l/m/c*, calculated the sum of the weights of the edges between them, and then counted the number of permutations, *k*, where the sum of weights was greater than or equal to that observed among the 22 candidate‐genes; then *P_emp_* = (*k* + 1)/100,001.

We obtained 229 genes whose orthologues’ disruption yields *hyperactivity* in mouse (termed “genes annotated with *hyperactivity*”; see Supplemental Information). To test if the 22 candidate‐genes were significantly co‐expressed in the human brain with these 229 genes, when compared to genes whose mouse orthologues are associated with other *l/m/c* phenotypes, we:

- Removed six genes present in both gene‐sets, (*GABRA5*, *MAPK3*, *ARX*, *LIMK1*, *RAI1*, and *RYR3*), and calculated the sum of the weights of the edges from the remaining 16 candidate‐genes to the remaining 223 genes annotated with hyperactivity in mouse.
- Obtained empirical 1‐sided *P*‐values from 100,000 permutations by picking at random 16 genes from 433 genes whose mouse orthologues are associated with *l/m/c* phenotypes (six genes excluded in step (i) were also excluded here); then calculating the sum of the weights of the edges from the randomly chosen genes to the 223 genes annotated with hyperactivity.

We repeated this analysis for 13 and 12 candidate‐genes from the ADHD‐meta and Hyper/SAS cohorts, respectively. We also repeated the analysis for 56 genes that were duplicated among *de novo* CNVs present in the genomes of 303 individuals with ID (but not *Hyperactivity/SAS*, autism or seizures; obtained from DECIPHER), and whose orthologues’ disruption yields an *l/m/c* phenotype in mouse (termed “ID‐cohort *l/m/c*‐genes”).

Finally, to test if the genes annotated with *hyperactivity* were more connected to the candidate‐genes than to the ID‐cohort *l/m/c*‐genes we repeated step (i), above, then obtained empirical 1‐sided *P*‐values from 100000 permutations by:

- Picking at random 16 genes from a set of 66 genes comprised of 16 candidate‐genes and 54 ID‐cohort *l/m/c*­‐genes not annotated with *hyperactivity* (four genes overlapped). (The six genes excluded in step (i) were also excluded here).
- Calculating the sum of the weights of the edges from the randomly chosen genes to the 223 genes annotated with *hyperactivity*.

## RESULTS

### Behavioural Phenotypes Are Enriched Among Genes That Are Overlapped by Gains in Patients With ADHD

We sought to uncover molecular pathways whose genes were disrupted by CNVs in ADHD. Combining data pertaining to cohorts published in three studies (“Elia cohort” [Elia et al., 2010], “Williams cohort” [Williams et al., 2010], and “Lionel cohort” [Lionel et al., 2011]; see Materials and Methods and Supplemental Table SI), we considered those genes overlapped by rare, predominantly inherited CNVs (“CNV‐genes”) in 902 patients with ADHD (the “ADHD‐meta cohort”).

We restricted our analysis to those CNVs > 500 Kb because (i) large, rare CNVs have been implicated in several other neurodevelopmental disorders (including intellectual disability (ID) [de Vries et al., 2005; Sharp et al., 2006], autism [Sebat et al., 2007; Marshall et al., 2008], schizophrenia and bipolar disorder [International Schizophrenia Consortium, 2008; Walsh et al., 2008; Grozeva et al., 2010]); (ii) CNVs in this size‐range have the greatest burden in ADHD cases compared to controls [Williams et al., 2010; Stergiakouli et al., 2012; Williams et al., 2012]; and (iii), very large variants are likely to be the most penetrant [Girirajan et al., 2011], and thus may causally contribute to the disorder. Sixty‐seven individuals contributed 71 CNVs > 500 Kb (Table IA). After removing genes overlapped by common CNVs in healthy individuals and thus unlikely to be particularly penetrant [Shaikh et al., 2009] (see Materials and Methods and Table IC), we retained 244 “gain‐genes” and 204 “loss‐genes” (genes overlapped by gains and losses, respectively; see Materials and Methods and Table IIA).

We then examined whether the ADHD CNV‐genes were enriched in genes whose orthologues’ mouse models were associated with particular phenotypes. As has previously been demonstrated in analyses of behavioral disorders, mouse phenotypes are informative for the analysis of human behavioral disorders because they capture complex system properties such as behavior better than more molecular gene annotations [Webber et al., 2009; Elia et al., 2010; Noh et al. 2013]. We employed mouse phenotype data from the Mouse Genome Informatics (MGI) resource (http://www.informatics.jax.org [Eppig et al., 2007]) to annotate the CNV‐genes (see Materials and Methods). In the ADHD‐meta cohort, 58/244 (24%) gain‐genes and 48/204 (24%) loss‐genes had orthologous genes whose disruption yields phenotypes in mouse (Table IIA).

Since ADHD is a behavioral disorder, we tested the ADHD‐meta cohort for an enrichment of CNV‐genes whose orthologues were associated with mouse phenotypes classed as “*Behaviour/neurological*” within the MGI annotations. We found that *Behavioural/neurological* phenotypes were enriched among the mouse orthologues of gain‐genes (28/58 (48%) genes, 1.5‐fold enrichment, *P* = 0.01), but not among the orthologues of loss‐genes (12/48 (25%) genes, 0.76‐fold change, *P* = 0.9). To refine the enrichment identified among the gain‐genes, we then tested 158 more specific *Behaviour/neurological* phenotypes (see Materials and Methods, Supplemental Information and Supplemental Figure S2). At an FDR of 5%, only genes whose orthologues’ disruption yields an *abnormal learning/memory/conditioning* (*l/m/c*) phenotype in mouse were significantly enriched (13/58 (22%) genes, 3.2‐fold enrichment, *P* = 1 × 10^−4^). Gain‐genes whose orthologues’ disruption yields this phenotype were present in 11/67 (16%) of individuals with CNVs > 500 Kb (Fig. 1A). We verified that the observed functional enrichments among the mouse orthologues of gain‐genes were not caused by a length bias in the genes (see Supplemental Information). As a control experiment, we repeated the analysis using a cohort of healthy individuals [Shaikh et al., 2009]: among 90 genes which were overlapped by 71 rare gains >500 Kb and whose mouse orthologues had associated phenotypes, we found no significant enrichment of genes whose orthologues’ disruption yields *l/m/*c in mouse (10/90 (11%) genes, 1.6‐fold enrichment, *P *> 0.05).

### Attempted Replication in a Second ADHD Cohort

While this study was underway, three further data sets of rare CNVs in ADHD patients were published, including 1,842 individuals with ADHD not included in the ADHD meta‐cohort [Jarick et al., 2014; Stergiakouli et al., 2012; Williams et al., 2012] (Supplemental Table SI). Combining these new cohorts into the “ADHD‐replication cohort”, we identified 537 gain‐genes affected by large gains (>500 Kb) in up to 180 patients (Tables IB and IIB), and which were not observed in common gains among controls. Among those 144 (27%) gain‐genes whose mouse orthologues had associated phenotypes (Table IIB), there was no significant enrichment of genes whose orthologues’ disruption yields *l/m/*c in mouse (8/144 (6%) gain‐genes associated with *l/m/c*, 0.8‐fold change, *P* = 0.8). Among the combined set of 192 analyzable gain‐genes, arising in either the ADHD‐meta or ADHD‐replication cohorts, there was a significant enrichment of genes whose orthologues’ disruption yields an *l/m/c* phenotype in mouse (21/192 (11%) genes, 1.6‐fold enrichment, *P* = 0.02). However, this enrichment is largely formed by the first ADHD meta‐cohort.

### Gain‐Genes in Patients With Hyperactivity and/or Short Attention Span Are Associated With Abnormal Learning/Memory/Conditioning

Next, we used the DECIPHER database [Firth et al., 2009] to obtain CNVs present in individuals with ADHD‐related human phenotypes (see Materials and Methods and Supplemental Information). DECIPHER records genotypic and phenotypic data on individuals with neurodevelopmental disorders and reports individual clinical phenotypes using terms defined by the London Dysmorphology Database (LDD, [Fryns and de Ravel, 2002]), wherein there is no single term that directly describes ADHD. However, by mapping LDD terms to the Human Phenotype Ontology (HPO, [Robinson and Mundlos, 2010]), we see that within DECIPHER an individual with ADHD must be described using the LDD terms *Hyperactivity* and/or *Short attention span* (abbreviated to “*Hyperactivity/SAS*”; see Materials and Methods and Supplemental Figure S3). Therefore, we identified a cohort of 22 individuals with Hyperactivity/*SAS* (herein termed the “Hyper/SAS cohort”), and whose genomes harbored at least one *de novo* CNV (Supplemental Table SII). We selected only *de novo*, rather than inherited, CNVs as these are more likely to be causal in these patients’ prominent neurodevelopmental disorders and because of the variable reporting of inherited CNVs within DECIPHER [Stankiewicz and Lupski, 2010; Veltman and Brunner, 2012]. In accordance with the studies providing our ADHD‐meta cohort [Elia et al., 2010; Williams et al., 2010; Lionel et al., 2011], we selected the cohort so that no individual presented phenotypes associated with autism or seizures; however, 21/22 individuals in the Hyper/SAS cohort had ID (see Supplemental Information; we return to address this later).

Filtering out CNVs < 500 Kb from the Hyper/SAS cohort left eight gains in eight individuals (see Materials and Methods and Supplemental Table SII), overlapping 166 genes not also present in common gains among controls. Among the 55/166 (33%) gain‐genes for which orthologous mouse models and corresponding phenotypes were available, 12 (22%) had orthologues whose disruption yields *l/m/c* in mouse (3.2‐fold enrichment, *P* = 3 × 10^−4^). Nine of the twelve gain‐genes were not among those identified in the ADHD‐meta cohort (Fig. 1B), whereas the five individuals whose gains harbored these genes were drawn from all three contributing phenotype groups (*Hyperactivity*‐only, *SAS*‐only, and *Hyperactivity* with *SAS* (Supplemental Table SIII)).

### Gain‐Genes Associated With Abnormal Learning/Memory/Conditioning Are Co‐Expressed in the Human Brain

The phenotype *l/m/c* that was enriched among the ADHD‐meta and Hyper/SAS gain‐genes is a generalized mouse phenotype (encompassing 38 more specific phenotypes (Supplemental Figure S2)) that has been observed in the mouse models of 439 genes; only a fraction of these genes’ orthologues may causally contribute to ADHD. Consequently, we hypothesized that the 22 genes that formed our enrichments (“candidate‐genes”) might participate in shared biological processes in humans; moreover, that these biological processes are specific to this set of genes, and thus to ADHD and ADHD‐related phenotypes of *Hyperactivity/SAS*, as compared to random sets of genes whose mouse orthologues are associated with *l/m/c*.

**Figure 2:**
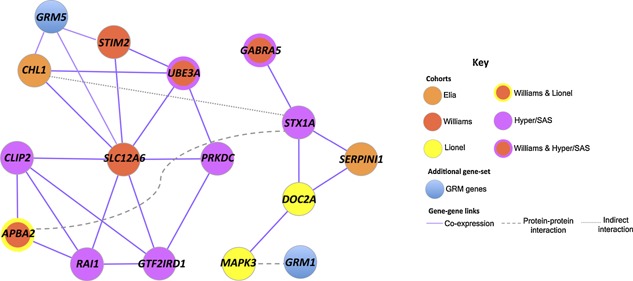
Gain‐genes whose 1:1 mouse orthologues’ disruption yields *abnormal learning/memory/conditioning* are expressed together in human brain. Network of co‐expression, in human brain, among 14 candidate‐genes from the ADHD‐meta and Hyper/SAS cohorts. Genes are drawn as circles and colored by cohort according to the key shown in the figure, and unbroken purple lines connect co‐expressed genes. We also show how this network overlaps with an ADHD‐associated glutamatergic network [Elia et al., 2012]: genes co‐expressed with *GRM5* are connected to the gene by unbroken purple lines, and a protein‐protein interaction between the protein products of *GRM5* and *MAPK3* is depicted with a dashed gray line. Finally, we have annotated the co‐expression network with protein‐protein interaction data and indirect interaction data; dashed gray lines connect pairs of candidate‐genes whose protein products interact, and dotted gray lines connect genes with indirect interactions.

To address this, we built a human gene co‐expression network using spatial and temporal maps of gene expression in the human brain available from BrainSpan ([Allen Institute for Brain Science], see Materials and Methods). In this network, the connection between two genes corresponds to the similarity in their brain expression patterns. We found that the 22 candidate‐genes were significantly more co‐expressed than random sets of 22 genes whose orthologues’ disruption yields *l/m/c* in mouse (*P* = 0.014; 14/22 candidate‐genes participate in the identified co‐expression network (Fig. 2); see Materials and Methods). Thus, the majority of these candidate‐genes form a sub‐network of genes that are tightly co‐expressed within the brain, as compared to random genes with *l/m/c* associations. We asked if the co‐expression network was primarily composed of co‐expressed genes that were also co‐localized to the same chromosomal region; it was not, with only 2/21 (10%) of pairs of co‐expressed genes affected by the same gain CNV (gene‐pairs *CLIP2* & *GTF2IRD1*, and *DOC2A* & *MAPK3*; see Figs. 1B and Fig. 2).

As all of the individuals in the Hyper/SAS cohort also presented with ID, any functional enrichment among genes affected by CNVs in this cohort might be associated with these individuals’ ID phenotype rather than their ADHD‐related phenotypes. To address this concern, we obtained a cohort of 303 individuals from DECIPHER who had ID but not *Hyperactivity/SAS*, autism or seizures, and from that cohort identified 56 genes duplicated among *de novo* CNVs and whose orthologues’ disruption yields an *l/m/c* phenotype in mouse (herein termed “ID‐cohort *l/m/c*‐genes”). We found that the ADHD‐meta cohort candidate‐genes were significantly more connected to the DECIPHER Hyper/SAS candidate‐genes than to the DECIPHER ID‐cohort *l/m/c*‐genes (*P* = 4 × 10^−3^; see Supplemental Information). This suggests that the *l/m/c* enrichment among the mouse orthologues of Hyper/SAS cohort gain‐genes was related to these individuals’ ADHD‐related phenotypes.

### ADHD and Hyperactivity/SAS Candidate‐Genes Are Co‐Expressed With Genes Whose Mouse Orthologues Are Associated With an ADHD Face‐Valid Phenotype

Previous work has suggested that face‐valid mouse phenotypes for ADHD are hyperactivity, reduced attention, and impulsivity [Bruno et al., 2007]. The three MGI mouse phenotypes that correspond to these human phenotypes are, respectively: *hyperactivity*, *abnormal latent inhibition of conditioning behavior*, and *abnormal impulsive behavior control*. We focused our analysis on just the set of 229 human genes whose orthologue's disruption yields *hyperactivity* in the mouse (“genes annotated with hyperactivity”; see Supplemental Information) because only six and four genes were annotated with the other two phenotypes, respectively.

We asked whether the 22 candidate‐genes were more tightly co‐expressed in the human brain with genes whose mouse orthologues are associated with *hyperactivity*, as compared to other genes whose mouse orthologues are associated with *l/m/c* phenotypes. The mouse orthologues of 6 of the 22 candidate‐genes are associated with *hyperactivity* and for the purpose of this analysis were removed from both sets of genes. Indeed, we found that the remaining 16 candidate‐genes were significantly more connected to genes annotated with *hyperactivity* than were genes whose mouse orthologues are associated with the more general *l/m/c* phenotype (*P* = 7 × 10^−3^; Supplemental Figure S6; see Materials and Methods). Genes found in both the ADHD‐meta cohort and the Hyper/SAS cohort contributed to the connections (Table III and Supplemental Figure S6). The 13 candidate‐genes from the ADHD‐meta cohort were significantly connected to the set of genes annotated with *hyperactivity* in mouse (*P* = 0.02), even when the candidate‐genes only found in the Hyper/SAS cohort were excluded. Although the connections between the 12 Hyper/SAS‐cohort candidate‐genes and genes annotated with *hyperactivity* was not significant alone (*P* = 0.06), they contributed to the increased significance reported for the combined analyses.

**Table III:** Co‐Expression Gene‐Pairs Between Candidate‐Genes, and Genes Whose Orthologues’ Disruption Yields *hyperactivity* in Mouse

Cohort	Number of cohort candidate‐genes co‐expressed with genes annotated with *hyperactivity*	Number of co‐expressed gene‐pairs between cohort candidate‐genes and genes annotated with *hyperactivity*
ADHD‐meta	7	117
Hyper/SAS	4	80
Both	2	27

For each cohort we show the number of candidate‐genes co‐expressed with genes whose 1:1 orthologues’ disruption yields *hyperactivity* in mouse (“genes annotated with *hyperactivity*”), and then we show the number of co‐expressed gene‐pairs between the sets of genes. The last row of the table gives the statistics for the candidate‐genes that are present in both the ADHD‐meta and the Hyper/SAS cohorts.

Finally, we looked for evidence that the co‐expression of the ADHD candidate genes with hyperactivity genes was specific to ADHD rather than ID. For this, we asked whether the 56 DECIPHER ID‐cohort *l/m/c*‐genes were also significantly connected to the set of genes annotated with *hyperactivity* in mouse, as compared to all genes annotated with *l/m/c* in mouse (see Materials and Methods); they were not (*P* = 0.3). Crucially, we found that genes annotated with *hyperactivity* in mouse were significantly more connected to the ADHD candidate‐genes than to the DECIPHER ID‐cohort *l/m/c*‐genes (*P* = 0.02; see Materials and Methods).

## DISCUSSION

In this study, we explored the hypothesis that distinct CNVs give rise to ADHD by affecting genes participating in shared biological processes, the perturbation of which causes the disorder. Analyzing genes in duplications from individuals with ADHD and ADHD‐related phenotypes, we identified an enrichment of duplicated genes whose loss in the mouse is associated with *abnormal learning/memory/conditioning* phenotypes (*l/m/c*), yielding 22 candidate‐genes of interest. We found that these 22 *l/m/c* candidate‐genes are co‐expressed spatially and temporally within the human brain suggesting that they participate in shared biological processes. Finally, we found that the 22 candidate genes are co‐expressed with genes whose disruption is associated with *hyperactivity*, an integral phenotype in face‐valid mouse models of ADHD, and that this association is significantly stronger for these ADHD candidate genes as compared to genes similarly selected from CNVs identified within individuals with ID.

Our enrichment was found among gain‐genes, but the associated mouse models result from gene losses; moreover, we did not find a specific enrichment of *hyperactivity* phenotypes among gain‐genes, but of a more general *l/m/c* phenotype. These results are consistent with examples of genes whose deletions predispose to one set of neuropsychiatric disorders but whose duplications influence another. For example, the disruption of SHANK3 has been implicated ASD, ID, and SCZ whereas its over‐expression in the mouse causes manic‐like behavior [Bonaglia et al., 2006; Durand et al., 2007; Moessner et al., 2007; Gauthier et al., 2009; Gauthier et al., 2010; Grabrucker et al., 2011; Han et al., 2013]. Corroboratively, 11 of our 22 candidate‐genes have been implicated in ASD or SCZ or other neurological or neuropsychiatric syndromes by a variety of genetic variants (Supplemental Tables SIV and SV). It is likely, therefore, that some of these genes are dosage‐sensitive so that increased or decreased levels are deleterious [Gout et al., 2010], as has been shown for proteins at the synapse [Sugiyama et al., 2005].

We were unable to replicate the enrichment of *l/m/c*‐associated genes among the gain‐genes of a second, larger, cohort of ADHD patients, which may indicate that our initial result was a false positive. However, to argue against this, we highlight the concordances in gene expression patterns between the candidate genes similarly identified in the ADHD‐meta cohort and the DECIPHER Hyper/SAS cohort, and between these genes and those that influence hyperactivity phenotypes in the mouse. We note that mouse phenotypes have only been investigated for the orthologues of ~1/3rd of human genes, diminishing the power of our approach. Of these, the mouse orthologues of only 2,089 genes are annotated with *Behaviour/neurological* phenotypes and, more specifically, only 439 genes with an *l/m/c* phenotype. Furthermore, of the 67 patients considered in the ADHD‐meta cohort, our findings are able to provide a causal hypothesis for 11 (16%) patients (Fig. 1A). The unexplained 84% of patients may possess genetic variants of a type not considered in this study, and affecting genes that participate in the network reported here. However, it may also be that our study has instead alighted upon only one among multiple molecular mechanisms underlying ADHD that may not be similarly represented in other cohorts.

Nonetheless, the enrichment was also found among a set of *de novo‐*gain‐genes present in individuals with human phenotypes of *Hyperactivity* and *Short attention span* (*SAS*), obtained from DECIPHER. There are two considerations regarding the use of this cohort. The first is that all of the patients in the DECIPHER cohort also had ID and that any functional enrichment among genes affected by CNVs in this cohort might be associated with these individuals’ ID phenotype rather than their ADHD‐related phenotypes. The heterogeneity and complexity of ID and ADHD, and the potential impact that the disorders have on one another in individuals comorbid for them, means that we cannot rule this out. However, using our brain‐specific co‐expression network, we found evidence to suggest that the *l/m/c* enrichment among the mouse orthologues of Hyper/SAS cohort gain‐genes was related to these individuals’ ADHD‐related phenotypes. Moreover, recent work suggests that children with ADHD and mild ID are not a clinically distinct ADHD subgroup [Ahuja et al., 2013]. The second consideration regarding our use of this cohort is that *Hyperactivity* and *SAS* are not synonymous with ADHD; however, they are immediate ontological ancestors of ADHD, and any patient with ADHD recorded in DECIPHER must be described using these terms (Supplemental Figure S3). Moreover, we propose that if we are to establish a molecular basis for ADHD, then it is vital to study the shared processes among genes implicated in both ADHD and ADHD‐related phenotypes. The co‐expression of the two sets of candidate genes in the brain, and with genes whose orthologues’ disruption in the mouse yields hyperactivity phenotypes, supports a shared ADHD‐relevant molecular etiology for individuals from both the ADHD meta‐cohort and the Hyper/SAS cohort.

The identified co‐expression network included 14 of 22 candidate‐genes, comprising genes from both cohorts. We placed this co‐expression network in the context of previously identified ADHD‐associated glutamatergic and neurodevelopmental networks [Poelmans et al., 2011; Elia et al., 2012]. Three of the genes in the network (*CHL1*, *STIM2*, and *SLC12A6*) were co‐expressed with the metabotropic glutamate receptor *GRM5*, and *MAPK3* had a known protein‐protein interaction with another metabotropic glutamate receptor, *GRM1* (Fig. 2). In addition, two of the co‐expressed 14 genes, *MAPK3* and *SERPINI1*, participated in the neurodevelopmental network for ADHD proposed by Poelmans et al., [Poelmans et al., 2011]. We also annotated the co‐expression network with known protein‐protein and indirect gene‐gene interactions: the two clusters formed by the 14 candidate‐genes were connected by an interaction between the protein products of *STX1A* (part of the SNARE complex) and *APBA2* (Fig. 2). *APBA2* is part of a multi‐protein complex, which probably functions as an intermediate in neurotransmitter vesicle docking [Biederer and Sudhof, 2000; Dulubova et al., 2007; Kirov et al., 2008]; this complex also includes members of the SNARE complex. Additionally, in mouse brain, *CHL1* has been shown to have a role in the selective activation of the presynaptic machinery chaperoning the SNARE complex [Andreyeva et al., 2010], thus providing evidence of an indirect interaction between *CHL1* and *STX1A* (Fig. 2).

In conclusion, we have identified a previously unknown network of co‐expressed genes preferentially disrupted among patients with ADHD or ADHD‐related phenotypes, which both proposes a common molecular etiology and, if confirmed, provides targets for the development of therapeutic interventions.

## Supporting information

Additional supporting information may be found in the online version of this article at the publisher's web‐site.

Supporting Information.

## References

[ajmgb32285-bib-0001] Adamou M , Arif M , Asherson P , Aw TC , Bolea B , Coghill D , Guethjonsson G , Halmoy A , Hodgkins P , Muller U , et al. 2013 Occupational issues of adults with ADHD. BMC Psychiatry 13:59. 10.1186/1471-244X-13-59 PMC3599848 23414364

[ajmgb32285-bib-0002] Ahuja A , Martin J , Langley K , Thapar A . 2013 Intellectual disability in children with attention deficit hyperactivity disorder. J Pediatr 163(3):890–895 e891. 23608559 10.1016/j.jpeds.2013.02.043 PMC4078221

[ajmgb32285-bib-0003] Allen Institute for Brain Science. Downloaded 8th May 2012 BrainSpan: Atlas of the Developing Human Brain [http://www.brainspan.org].

[ajmgb32285-bib-0004] Andreyeva A , Leshchyns'ka I , Knepper M , Betzel C , Redecke L , Sytnyk V , Schachner M . 2010 CHL1 is a selective organizer of the presynaptic machinery chaperoning the SNARE complex. PLoS ONE 5(8):e12018. 10.1371/journal.pone.0012018 PMC2920317 20711454

[ajmgb32285-bib-0005] American Psychiatric Association. 2000 Diagnostic and statistical manual of mental disorders (4th ed., text rev.). Washington, DC: Author.

[ajmgb32285-bib-0006] Biederer T , Sudhof TC . 2000 Mints as adaptors. Direct binding to neurexins and recruitment of munc18. J Biol Chem 275(51):39803–39806. 11036064 10.1074/jbc.C000656200

[ajmgb32285-bib-0007] Biederman J , Wilens T , Mick E , Milberger S , Spencer TJ , Faraone SV . 1995 Psychoactive substance use disorders in adults with attention deficit hyperactivity disorder (ADHD): Effects of ADHD and psychiatric comorbidity. Am J Psychiatry 152(11):1652–1658. 7485630 10.1176/ajp.152.11.1652

[ajmgb32285-bib-0008] Bonaglia MC , Giorda R , Mani E , Aceti G , Anderlid BM , Baroncini A , Pramparo T , Zuffardi O . 2006 Identification of a recurrent breakpoint within the SHANK3 gene in the 22q13.3 deletion syndrome. J Med Genet 43(10):822–828. 16284256 10.1136/jmg.2005.038604 PMC2563164

[ajmgb32285-bib-0009] Bruno KJ , Freet CS , Twining RC , Egami K , Grigson PS , Hess EJ . 2007 Abnormal latent inhibition and impulsivity in coloboma mice, a model of ADHD. Neurobiol Dis 25(1):206–216. 17064920 10.1016/j.nbd.2006.09.009 PMC1761697

[ajmgb32285-bib-0010] Cross‐Disorder Group of the Psychiatric Genomics Consortium . 2013 Genetic relationship between five psychiatric disorders estimated from genome‐wide SNPs. Nat Genet 45(9):984–994. 23933821 10.1038/ng.2711 PMC3800159

[ajmgb32285-bib-0011] Danckaerts M , Sonuga‐Barke EJ , Banaschewski T , Buitelaar J , Dopfner M , Hollis C , Santosh P , Rothenberger A , Sergeant J , Steinhausen HC , et al. 2010 The quality of life of children with attention deficit/hyperactivity disorder: A systematic review. Eur Child Adolesc Psychiatry 19(2):83–105. 19633992 10.1007/s00787-009-0046-3 PMC3128746

[ajmgb32285-bib-0012] de Vries BB , Pfundt R , Leisink M , Koolen DA , Vissers LE , Janssen IM , Reijmersdal S , Nillesen WM , Huys EH , Leeuw N , et al. 2005 Diagnostic genome profiling in mental retardation. Am J Hum Genet 77(4):606–616. 16175506 10.1086/491719 PMC1275609

[ajmgb32285-bib-0013] Diagnostic and statistical manual of mental disorders.

[ajmgb32285-bib-0014] Dulubova I , Khvotchev M , Liu S , Huryeva I , Sudhof TC , Rizo J . 2007 Munc18–1 binds directly to the neuronal SNARE complex. Proc Natl Acad Sci U S A 104(8):2697–2702. 17301226 10.1073/pnas.0611318104 PMC1815244

[ajmgb32285-bib-0015] Durand CM , Betancur C , Boeckers TM , Bockmann J , Chaste P , Fauchereau F , Nygren G , Rastam M , Gillberg IC , Anckarsater H , et al. 2007 Mutations in the gene encoding the synaptic scaffolding protein SHANK3 are associated with autism spectrum disorders. Nat Genet 39(1):25–27. 17173049 10.1038/ng1933 PMC2082049

[ajmgb32285-bib-0016] Elia J , Gai X , Xie HM , Perin JC , Geiger E , Glessner JT , D'Arcy M , deBerardinis R , Frackelton E , Kim C , et al. 2010 Rare structural variants found in attention‐deficit hyperactivity disorder are preferentially associated with neurodevelopmental genes. Mol Psychiatry 15(6):637–646. 19546859 10.1038/mp.2009.57 PMC2877197

[ajmgb32285-bib-0017] Elia J , Glessner JT , Wang K , Takahashi N , Shtir CJ , Hadley D , Sleiman PM , Zhang H , Kim CE , Robison R , et al. 2012 Genome‐wide copy number variation study associates metabotropic glutamate receptor gene networks with attention deficit hyperactivity disorder. Nat Genet 44(1):78–84. 22138692 10.1038/ng.1013 PMC4310555

[ajmgb32285-bib-0018] Eppig JT , Blake JA , Bult CJ , Richardson JE , Kadin JA , Ringwald M , staff MGI . 2007 Mouse genome informatics (MGI) resources for pathology and toxicology. Toxicol Pathol 35(3):456–457. 17474068 10.1080/01926230701310536

[ajmgb32285-bib-0019] Faraone SV , Perlis RH , Doyle AE , Smoller JW , Goralnick JJ , Holmgren MA , Sklar P . 2005 Molecular genetics of attention‐deficit/hyperactivity disorder. Biol Psychiatry 57(11):1313–1323. 15950004 10.1016/j.biopsych.2004.11.024

[ajmgb32285-bib-0020] Fayyad J , De Graaf R , Kessler R , Alonso J , Angermeyer M , Demyttenaere K , De Girolamo G , Haro JM , Karam EG , Lara C , et al. 2007 Cross‐national prevalence and correlates of adult attention‐deficit hyperactivity disorder. Br J Psychiatry 190:402–409. 17470954 10.1192/bjp.bp.106.034389

[ajmgb32285-bib-0021] Firth HV , Richards SM , Bevan AP , Clayton S , Corpas M , Rajan D , Van Vooren S , Moreau Y , Pettett RM , Carter NP . 2009 DECIPHER: Database of chromosomal imbalance and phenotype in humans using ensembl resources. Am J Hum Genet 84(4):524–533. 19344873 10.1016/j.ajhg.2009.03.010 PMC2667985

[ajmgb32285-bib-0022] Franke B , Neale BM , Faraone SV . 2009 Genome‐wide association studies in ADHD. Hum Genet 126(1):13–50. 19384554 10.1007/s00439-009-0663-4 PMC3774416

[ajmgb32285-bib-0023] Fryns JP , de Ravel TJ . 2002 London Dysmorphology Database, London Neurogenetics Database and Dysmorphology Photo Library on CD‐ROM [Version 3] 2001R. M. Winter, M. Baraitser, Oxford University Press, ISBN 019851–780, pound sterling 1595. Hum Genet 111(1):113.

[ajmgb32285-bib-0024] Gauthier J , Champagne N , Lafreniere RG , Xiong L , Spiegelman D , Brustein E , Lapointe M , Peng H , Cote M , Noreau A , et al. 2010 De novo mutations in the gene encoding the synaptic scaffolding protein SHANK3 in patients ascertained for schizophrenia. Proc Natl Acad Sci U S A 107(17):7863–7868. 20385823 10.1073/pnas.0906232107 PMC2867875

[ajmgb32285-bib-0025] Gauthier J , Spiegelman D , Piton A , Lafreniere RG , Laurent S , St‐Onge J , Lapointe L , Hamdan FF , Cossette P , Mottron L , et al. 2009 Novel de novo SHANK3 mutation in autistic patients. Am J Med Genet B Neuropsychiatr Genet 150B(3):421–424. 18615476 10.1002/ajmg.b.30822

[ajmgb32285-bib-0026] Girirajan S , Brkanac Z , Coe BP , Baker C , Vives L , Vu TH , Shafer N , Bernier R , Ferrero GB , Silengo M , et al. 2011 Relative burden of large CNVs on a range of neurodevelopmental phenotypes. PLoS Genet 7(11):e1002334. 10.1371/journal.pgen.1002334 PMC3213131 22102821

[ajmgb32285-bib-0027] Gout JF , Kahn D , Duret L , Paramecium Post‐Genomics . 2010 The relationship among gene expression, the evolution of gene dosage, and the rate of protein evolution. PLoS Genet 6(5):e1000944. 10.1371/journal.pgen.1000944 PMC2869310 20485561

[ajmgb32285-bib-0028] Grabrucker AM , Schmeisser MJ , Schoen M , Boeckers TM . 2011 Postsynaptic ProSAP/Shank scaffolds in the cross‐hair of synaptopathies. Trends Cell Biol 21(10):594–603. 21840719 10.1016/j.tcb.2011.07.003

[ajmgb32285-bib-0029] Grozeva D , Kirov G , Ivanov D , Jones IR , Jones L , Green EK , St Clair DM , Young AH , Ferrier N , Farmer AE , et al. 2010 Rare copy number variants: A point of rarity in genetic risk for bipolar disorder and schizophrenia. Arch Gen Psychiatry 67(4):318–327. 20368508 10.1001/archgenpsychiatry.2010.25 PMC4476027

[ajmgb32285-bib-0030] Hamshere ML , Langley K , Martin J , Agha SS , Stergiakouli E , Anney RJ , Buitelaar J , Faraone SV , Lesch KP , Neale BM , et al. 2013 High loading of polygenic risk for ADHD in children with comorbid aggression. Am J Psychiatry 170(8):909–916. 23599091 10.1176/appi.ajp.2013.12081129 PMC3935265

[ajmgb32285-bib-0031] Han K , Holder JL Jr. , Schaaf CP , Lu H , Chen H , Kang H , Tang J , Wu Z , Hao S , Cheung SW , et al. 2013 SHANK3 overexpression causes manic‐like behaviour with unique pharmacogenetic properties. Nature 503(7474):72–77. 24153177 10.1038/nature12630 PMC3923348

[ajmgb32285-bib-0032] Hinrichs AS , Karolchik D , Baertsch R , Barber GP , Bejerano G , Clawson H , Diekhans M , Furey TS , Harte RA , Hsu F , et al. 2006 The UCSC genome browser database: Update 2006. Nucleic Acids Res 34(Database issue):D590–D598. 16381938 10.1093/nar/gkj144 PMC1347506

[ajmgb32285-bib-0033] International Schizophrenia Consortium . 2008 Rare chromosomal deletions and duplications increase risk of schizophrenia. Nature 455(7210):237–241. 18668038 10.1038/nature07239 PMC3912847

[ajmgb32285-bib-0034] Storey. 2002 A direct approach to false discovery rates. Journal of the Royal Statistical Society. Series B 64(3):479–498.

[ajmgb32285-bib-0035] Jarick I , Volckmar AL , Putter C , Pechlivanis S , Nguyen TT , Dauvermann MR , Beck S , Albayrak O , Scherag S , Gilsbach S , et al. 2014 Genome‐wide analysis of rare copy number variations reveals PARK2 as a candidate gene for attention‐deficit/hyperactivity disorder. Mol Psychiatry 19(1):115–121. 23164820 10.1038/mp.2012.161 PMC3873032

[ajmgb32285-bib-0036] Kessler RC , Adler L , Barkley R , Biederman J , Conners CK , Demler O , Faraone SV , Greenhill LL , Howes MJ , Secnik K , et al. 2006 The prevalence and correlates of adult ADHD in the United States: Results from the National Comorbidity Survey Replication. Am J Psychiatry 163(4):716–723. 16585449 10.1176/appi.ajp.163.4.716 PMC2859678

[ajmgb32285-bib-0037] Kirov G , Gumus D , Chen W , Norton N , Georgieva L , Sari M , O'Donovan MC , Erdogan F , Owen MJ , Ropers HH , et al. 2008 Comparative genome hybridization suggests a role for NRXN1 and APBA2 in schizophrenia. Hum Mol Genet 17(3):458–465. 17989066 10.1093/hmg/ddm323

[ajmgb32285-bib-0038] Kuntsi J , Neale BM , Chen W , Faraone SV , Asherson P . 2006 The IMAGE project: Methodological issues for the molecular genetic analysis of ADHD. Behav Brain Funct 2:27. 10.1186/1744-9081-2-27 PMC1559631 16887023

[ajmgb32285-bib-0039] Langfelder P , Horvath S . 2008 WGCNA: An R package for weighted correlation network analysis. BMC Bioinformatics 9:559. 10.1186/1471-2105-9-559 PMC2631488 19114008

[ajmgb32285-bib-0040] Larson K , Russ SA , Kahn RS , Halfon N . 2011 Patterns of comorbidity, functioning, and service use for US children with ADHD, 2007. Pediatrics 127(3):462–470. 21300675 10.1542/peds.2010-0165 PMC3065146

[ajmgb32285-bib-0041] Lasky‐Su J , Neale BM , Franke B , Anney RJ , Zhou K , Maller JB , Vasquez AA , Chen W , Asherson P , Buitelaar J , et al. 2008 Genome‐wide association scan of quantitative traits for attention deficit hyperactivity disorder identifies novel associations and confirms candidate gene associations. Am J Med Genet B Neuropsychiatr Genet 147B(8):1345–1354. 18821565 10.1002/ajmg.b.30867

[ajmgb32285-bib-0042] Lesch KP , Timmesfeld N , Renner TJ , Halperin R , Roser C , Nguyen TT , Craig DW , Romanos J , Heine M , Meyer J , et al. 2008 Molecular genetics of adult ADHD: converging evidence from genome‐wide association and extended pedigree linkage studies. J Neural Transm 115(11):1573–1585. 18839057 10.1007/s00702-008-0119-3

[ajmgb32285-bib-0043] Lionel AC , Crosbie J , Barbosa N , Goodale T , Thiruvahindrapuram B , Rickaby J , Gazzellone M , Carson AR , Howe JL , Wang Z , et al. 2011 Rare copy number variation discovery and cross‐disorder comparisons identify risk genes for ADHD. Sci Transl Med 3(95):‐95ra75. 10.1126/scitranslmed.3002464 21832240

[ajmgb32285-bib-0044] Marshall CR , Noor A , Vincent JB , Lionel AC , Feuk L , Skaug J , Shago M , Moessner R , Pinto D , Ren Y , et al. 2008 Structural variation of chromosomes in autism spectrum disorder. Am J Hum Genet 82(2):477–488. 18252227 10.1016/j.ajhg.2007.12.009 PMC2426913

[ajmgb32285-bib-0045] Mick E , Todorov A , Smalley S , Hu X , Loo S , Todd RD , Biederman J , Byrne D , Dechairo B , Guiney A , et al. 2010 Family‐based genome‐wide association scan of attention‐deficit/hyperactivity disorder. J Am Acad Child Adolesc Psychiatry 49(9):898–905 e893. 20732626 10.1016/j.jaac.2010.02.014 PMC3730251

[ajmgb32285-bib-0046] Moessner R , Marshall CR , Sutcliffe JS , Skaug J , Pinto D , Vincent J , Zwaigenbaum L , Fernandez B , Roberts W , Szatmari P , et al. 2007 Contribution of SHANK3 mutations to autism spectrum disorder. Am J Hum Genet 81(6):1289–1297. 17999366 10.1086/522590 PMC2276348

[ajmgb32285-bib-0047] Neale BM , Lasky‐Su J , Anney R , Franke B , Zhou K , Maller JB , Vasquez AA , Asherson P , Chen W , Banaschewski T , et al. 2008 Genome‐wide association scan of attention deficit hyperactivity disorder. Am J Med Genet B Neuropsychiatr Genet 147B(8):1337–1344. 18980221 10.1002/ajmg.b.30866 PMC2831205

[ajmgb32285-bib-0048] Neale BM , Medland S , Ripke S , Anney RJ , Asherson P , Buitelaar J , Franke B , Gill M , Kent L , Holmans P , et al. 2010 Case‐control genome‐wide association study of attention‐deficit/hyperactivity disorder. J Am Acad Child Adolesc Psychiatry 49(9):906–920. 20732627 10.1016/j.jaac.2010.06.007 PMC2928577

[ajmgb32285-bib-0049] Noh HJ , Ponting CP , Boulding HC , Meader S , Betancur C , Buxbaum JD , Pinto D , Marshall CR , Lionel AC , Scherer SW , et al. 2013 Network topologies and convergent aetiologies arising from deletions and duplications observed in individuals with autism. PLoS Genet 9(6):e1003523. 10.1371/journal.pgen.1003523 PMC3675007 23754953

[ajmgb32285-bib-0050] Ohlmeier MD , Peters K , Te Wildt BT , Zedler M , Ziegenbein M , Wiese B , Emrich HM , Schneider U . 2008 Comorbidity of alcohol and substance dependence with attention‐deficit/hyperactivity disorder (ADHD). Alcohol Alcohol 43(3):300–304. 18326548 10.1093/alcalc/agn014

[ajmgb32285-bib-0051] Pelham WE , Foster EM , Robb JA . 2007 The economic impact of attention‐deficit/hyperactivity disorder in children and adolescents. J Pediatr Psychol 32(6):711–727. 17556402 10.1093/jpepsy/jsm022

[ajmgb32285-bib-0052] Poelmans G , Pauls DL , Buitelaar JK , Franke B . 2011 Integrated genome‐wide association study findings: Identification of a neurodevelopmental network for attention deficit hyperactivity disorder. Am J Psychiatry 168(4):365–377. 21324949 10.1176/appi.ajp.2010.10070948

[ajmgb32285-bib-0053] Polanczyk G , de Lima MS , Horta BL , Biederman J , Rohde LA . 2007 The worldwide prevalence of ADHD: A systematic review and metaregression analysis. Am J Psychiatry 164(6):942–948. 17541055 10.1176/ajp.2007.164.6.942

[ajmgb32285-bib-0054] Robinson PN , Mundlos S . 2010 The human phenotype ontology. Clin Genet 77(6):525–534. 20412080 10.1111/j.1399-0004.2010.01436.x

[ajmgb32285-bib-0055] Schachar R , Tannock R . 1995 Test of four hypotheses for the comorbidity of attention‐deficit hyperactivity disorder and conduct disorder. J Am Acad Child Adolesc Psychiatry 34(5):639–648. 7775359 10.1097/00004583-199505000-00016

[ajmgb32285-bib-0056] Sebat J , Lakshmi B , Malhotra D , Troge J , Lese‐Martin C , Walsh T , Yamrom B , Yoon S , Krasnitz A , Kendall J , et al. 2007 Strong association of de novo copy number mutations with autism. Science 316(5823):445–449. 17363630 10.1126/science.1138659 PMC2993504

[ajmgb32285-bib-0057] Shaikh TH , Gai X , Perin JC , Glessner JT , Xie H , Murphy K , O'Hara R , Casalunovo T , Conlin LK , D'Arcy M , et al. 2009 High‐resolution mapping and analysis of copy number variations in the human genome: A data resource for clinical and research applications. Genome Res 19(9):1682–1690. 19592680 10.1101/gr.083501.108 PMC2752118

[ajmgb32285-bib-0058] Sharp AJ , Hansen S , Selzer RR , Cheng Z , Regan R , Hurst JA , Stewart H , Price SM , Blair E , Hennekam RC , et al. 2006 Discovery of previously unidentified genomic disorders from the duplication architecture of the human genome. Nat Genet 38(9):1038–1042. 16906162 10.1038/ng1862

[ajmgb32285-bib-0059] Smith CL , Eppig JT . 2009 The mammalian phenotype ontology: Enabling robust annotation and comparative analysis. Wiley Interdiscip Rev Syst Biol Med 1(3):390–399. 20052305 10.1002/wsbm.44 PMC2801442

[ajmgb32285-bib-0060] Spencer TJ , Biederman J , Mick E . 2007 Attention‐deficit/hyperactivity disorder: Diagnosis, lifespan, comorbidities, and neurobiology. J Pediatr Psychol 32(6):631–642. 17556405 10.1093/jpepsy/jsm005

[ajmgb32285-bib-0061] Stankiewicz P , Lupski JR . 2010 Structural variation in the human genome and its role in disease. Annu Rev Med 61:437–455. 20059347 10.1146/annurev-med-100708-204735

[ajmgb32285-bib-0062] Stergiakouli E , Hamshere M , Holmans P , Langley K , Zaharieva I , de CG , Psychiatric GC , Hawi Z , Kent L , Gill M , et al. 2012 Investigating the contribution of common genetic variants to the risk and pathogenesis of ADHD. Am J Psychiatry 169(2):186–194. 22420046 10.1176/appi.ajp.2011.11040551 PMC3601404

[ajmgb32285-bib-0063] Sugiyama Y , Kawabata I , Sobue K , Okabe S . 2005 Determination of absolute protein numbers in single synapses by a GFP‐based calibration technique. Nat Methods 2(9):677–684. 16118638 10.1038/nmeth783

[ajmgb32285-bib-0064] Thapar A , Harrington R , McGuffin P . 2001 Examining the comorbidity of ADHD‐related behaviours and conduct problems using a twin study design. Br J Psychiatry 179:224–229. 11532799 10.1192/bjp.179.3.224

[ajmgb32285-bib-0065] Veltman JA , Brunner HG . 2012 De novo mutations in human genetic disease. Nat Rev Genet 13(8):565–575. 22805709 10.1038/nrg3241

[ajmgb32285-bib-0066] Wahlstedt C , Thorell LB , Bohlin G . 2009 Heterogeneity in ADHD: Neuropsychological pathways, comorbidity and symptom domains. J Abnorm Child Psychol 37(4):551–564. 19016322 10.1007/s10802-008-9286-9

[ajmgb32285-bib-0067] Walsh T , McClellan JM , McCarthy SE , Addington AM , Pierce SB , Cooper GM , Nord AS , Kusenda M , Malhotra D , Bhandari A , et al. 2008 Rare structural variants disrupt multiple genes in neurodevelopmental pathways in schizophrenia. Science 320(5875):539–543. 18369103 10.1126/science.1155174

[ajmgb32285-bib-0068] Webber C . 2011 Functional enrichment analysis with structural variants: Pitfalls and strategies. Cytogenet Genome Res 135(3–4):277–285. 21997137 10.1159/000331670

[ajmgb32285-bib-0069] Webber C , Hehir‐Kwa JY , Nguyen DQ , de Vries BB , Veltman JA , Ponting CP . 2009 Forging links between human mental retardation‐associated CNVs and mouse gene knockout models. PLoS Genet 5(6):e1000531. 10.1371/journal.pgen.1000531 PMC2694283 19557186

[ajmgb32285-bib-0070] Williams NM , Franke B , Mick E , Anney RJ , Freitag CM , Gill M , Thapar A , O'Donovan MC , Owen MJ , Holmans P , et al. 2012 Genome‐wide analysis of copy number variants in attention deficit hyperactivity disorder: The role of rare variants and duplications at 15q13.3. Am J Psychiatry 169(2):195–204. 22420048 10.1176/appi.ajp.2011.11060822 PMC3601405

[ajmgb32285-bib-0071] Williams NM , Zaharieva I , Martin A , Langley K , Mantripragada K , Fossdal R , Stefansson H , Stefansson K , Magnusson P , Gudmundsson OO , et al. 2010 Rare chromosomal deletions and duplications in attention‐deficit hyperactivity disorder: A genome‐wide analysis. Lancet 376(9750):1401–1408. 20888040 10.1016/S0140-6736(10)61109-9 PMC2965350

[ajmgb32285-bib-0072] Yang L , Neale BM , Liu L , Lee SH , Wray NR , Ji N , Li H , Qian Q , Wang D , Li J , et al. 2013 Polygenic transmission and complex neuro developmental network for attention deficit hyperactivity disorder: Genome‐wide association study of both common and rare variants. Am J Med Genet B Neuropsychiatr Genet 162B(5):419–430. 23728934 10.1002/ajmg.b.32169 PMC4321789

[ajmgb32285-bib-0073] Zhou K , Dempfle A , Arcos‐Burgos M , Bakker SC , Banaschewski T , Biederman J , Buitelaar J , Castellanos FX , Doyle A , Ebstein RP , et al. 2008 Meta‐analysis of genome‐wide linkage scans of attention deficit hyperactivity disorder. Am J Med Genet B Neuropsychiatr Genet 147B(8):1392–1398. 18988193 10.1002/ajmg.b.30878 PMC2890047

