# Supplementary material for: Duplications in ADHD patients harbour neurobehavioural genes that are co‐expressed with genes associated with hyperactivity in the mouse
Source: Am J Med Genet B Neuropsychiatr Genet. 2015 Feb 5;168(2):97–107. doi: 10.1002/ajmg.b.32285 (PMC4833176; doi:10.1002/ajmg.b.32285)
Supplement: Supplementary file 1 — Supporting Information. [file AJMG-168-97-s001.doc]

**Duplications in ADHD patients harbour neurobehavioural genes that are co-expressed with genes associated with hyperactivity in the mouse**

**Supplemental Information**

**Mammalian Phenotype Ontology *Behaviour/neurological phenotype* category: choosing which sub-phenotypes to test**

Phenotypes exhibited during mouse gene model experiments are described using the Mammalian Phenotype Ontology (MPO; ) and recorded in the Mouse Genome Informatics database (MGI; [http://www.informatics.jax.org](http://www.informatics.jax.org/) ; downloaded 16^th^ December 2011). There are 30 over-arching phenotypes in the MPO (**Supplemental Figure S2**); for our study, we focused on the *Behavioural/neurological* *phenotype* category. To reduce multiple-testing, we only considered the 159 sub-phenotypes in this category which annotated gene-sets containing >1% of all 2089 genes annotated with any *Behavioural/neurological* phenotype ; merging two phenotypes annotating identical gene-sets yielded 158 phenotypes to test.

**Functional enrichment: permutations**

We performed permutations to verify that the observed functional enrichments among CNV-genes were not caused by a length bias in the genes. To this end, we divided 6023 human protein-coding genes with Ensembl gene IDs whose orthologues’ disruption in mouse yields abnormal phenotypes into 100 bins *b_1_, b_2_, ... , b_100_* by their length according to Ensembl release 36. If the CNV-gene list had *g_1_, g_2_, ... , g_100_* genes from the bins *b_1_, b_2_, ..., b_100_* respectively, we obtained a random list by sampling *g_i_* genes from bin *b_i_*, *i* = 1, 2, ..., 100. Creating 100000 random lists matched by gene number and length to the CNV-gene list, we obtained empirical 1-sided *p*-values for the functional enrichments observed for in the CNV-gene list, by comparing their analytical *p*­-values, (calculated using a 1-sided hypergeometric test), to the analytical *p-*values calculated for the randomised gene-sets. Empirical *p-*values for functional enrichments were significant at 5% FDR.

**DECIPHER** **(DatabasE of Chromosomal Imbalance and Phenotype in Human using Ensembl Resources)**

DECIPHER is a database that contains clinical and CNV data for more than 2000 patients who present with multiple developmental disorders and congenital malformations (total 861 clinical phenotypes, median = 5, range = 1-32). Clinical phenotypes are described using terms from the London Dysmorphology Database (LDD, ). CNVs were detected via array comparative genomic hybridisation and categorised as one of *de-*novo, inherited, or having an unknown origin. Intellectual Disability (ID), termed *Mental retardation/developmental delay* in the database, is prevalent in 434/516 (84%) individuals who are recorded in the resource and have at least one *de* *novo* mutation.

**Resolving the issue of prevalence of ID in the Hyper/SAS cohort**

Among the gain-genes in the ADHD-meta and Hyper/SAS cohorts, 22 contributed to an enrichment of genes whose orthologues’ disruption yields *abnormal learning/memory/conditioning* (*l/m/c*) phenotypes in mouse (**Figure 1**). We refer to these 22 genes as “candidate-genes”. Thirteen candidate-genes were duplicated in patients in the ADHD-meta cohort, and 12 were duplicated in individuals from the Hyper/SAS cohort (3 gene overlap). As explained in the main text, all individuals in the Hyper/SAS cohort also had ID, so any functional enrichment among genes affected by CNVs in this cohort might be associated with these individuals’ ID phenotype rather than their ADHD-related phenotypes. We addressed this issue using the human brain-specific co-expression network described in **Methods and Materials**. To this end, we obtained a cohort of 303 individuals from DECIPHER who had ID but not *Hyperactivity/SAS*, autism or seizures, and from that cohort identified 56 genes duplicated among *de novo* CNVs and whose orthologues’ disruption yields an *l/m/c* phenotype in mouse (termed “ID-cohort *l/m/c*-genes”). Then we asked if the 13 ADHD-meta cohort candidate-genes were more connected to the 12 Hyper/SAS cohort candidate-genes than to the 56 ID-cohort *l/m/c*-genes. To answer this we:

1. Accounted for the overlap of 3 genes between the candidate-genes from the Hyper/SAS and ADHD-meta cohorts (**Figure 1B**): Treating these 3 genes as though they were present only in the ADHD-meta cohort, we calculated the sum of the edge-weights from the remaining 9 Hyper/SAS candidate-genes to the 13 ADHD-meta candidate-genes.
2. Obtained empirical 1-sided *P*-values (*P_emp_*): For 100000 permutations we randomly picked 9 genes from the 56 ID-cohort *l/m/c*-genes, calculated the sum of the weights of the edges from these genes to the 13 ADHD-meta cohort candidate-genes, and then counted the number of permutations, *k*, where the sum of the weights was greater than or equal to the sum of the weights observed from the 9 Hyper/SAS cohort candidate-genes to the ADHD-meta cohort candidate-genes; then *P_emp_* = (*k*+1)/100001.

**Additional phenotypes present in the Hyper/SAS cohort**

Including *Hyperactivity*, *Short attention span* (*SAS*) and *Mental retardation/ developmental delay* (*MR*, the LDD term for Intellectual Disability), there were 103 human phenotypes distributed among the 22 patients in the Hyper/SAS cohort (**Supplemental Figure S4A**), with the majority of patients (56%) diagnosed with between 5 and 10 phenotypes (**Supplemental Figure S4B**). However, 77 of the 100 phenotypes (excluding *Hyperactivity*, *SAS* and *MR*) were private to a single individual (**Supplemental Figure S4B**), and the two most common additional phenotypes, *Microcephaly* and *Speech Delay*, affected 6/22 (27%) individuals each. Consequently, we decided that the additional clinical phenotypes were unlikely to introduce generalised genetic enrichments (unrelated to *Hyperactivity*, *SAS* or *MR*).

**Obtaining genes whose orthologues’ disruption in mouse yields *hyperactivity***

Using the MGI, we obtained 254 human genes whose orthologues’ disruption yields *hyperactivity* in mouse. We excluded 25 genes whose mouse orthologues’ are also associated with the sub-phenotype *hyperactivity elicited by ethanol administration,* leaving a set of 229 genes with which to conduct our analyses (termed “genes annotated with *hyperactivity*”).

**BrainSpan gene co-expression networks: Sample brain regions**

We obtained normalised gene expression data from BrainSpan based on RNASeq of up to 16 brain regions from 41 individuals aged from 8 weeks post-conception to 40 years. Specifically, the brain regions included in the analysis were:

- Primary auditory cortex (core);
- Amygdaloid complex;
- Cerebellar cortex;
- Dorsolateral prefrontal cortex;
- Hippocampus;
- Posteroinferior (ventral) parietal cortex;
- Inferolateral temporal cortex (area TEv, area 20);
- Primary motor cortex (area M1, area 4);
- Mediodorsal nucleus of thalamus;
- Anterior (rostral) cingulate (medial prefrontal) cortex;
- Orbital frontal cortex;
- Primary somatosensory cortex (area S1, areas 3,1,2);
- Posterior (caudal) superior temporal cortex (area TAc);
- Striatum;
- Primary visual cortex (striate cortex, area V1/17);
- Ventrolateral prefrontal cortex.

**BrainSpan gene co-expression network results when the threshold for edge inclusion is relaxed**

To test if our results were robust to changes in the threshold on *r*, we repeated all of our analyses using the network composed of edges with weight *r* ≥0.5, and again with *r* ≥0.6. We found that: (i) our result showing connectivity of 22 candidate-genes in the BrainSpan gene-expression network, was robust to changes in threshold (*r* ≥0.5, *p*=0.049; *r* ≥0.6, *p*=0.051); (ii) our result showing that ADHD-meta candidate-genes were significantly more connected to Hyper/SAS candidate-genes than to 56 ID-cohort *l/m/c*-genes*,* was nearly robust to changes in threshold (*r* ≥0.5, *p*=0.064; *r* ≥0.6, *p*=0.054); (iii) our finding that ADHD and Hyper/SAS candidate-genes were significantly connected to a set of genes annotated with *hyperactivity* in mouse, was robust to changes in threshold (*r* ≥0.5, *p*=0.03; *r* ≥0.6, *p*=0.03), and; (iv) our finding that the genes annotated with *hyperactivity* were significantly more connected to the candidate-genes than to the DECIPHER ID-cohort *l/m/c*-genes, was affected by changes in threshold (*r* ≥0.5, *p*=0.1; *r* ≥0.6, *p*=0.1)

**Supplemental Tables**

**Supplemental Table SI**: Publicly available CNV data from seven cohorts of patients with ADHD

| Study | Platform (cases) | Date Published | Cases | Controls | Total rare CNVs (cases) | Total inherited CNVs (cases) |
| --- | --- | --- | --- | --- | --- | --- |
| **Elia *et al.* ^a^** | Illumina HumanHap550 BeadChip | 2010 | 335 | 2026 | 222 (≥10 SNPs) | 222 |
| **Williams *et al.* ^a^** | Illumina Human660W-Quad BeadChip | 2010 | 319 (excluding 33 with IQ < 70 and 14 with unknown IQ) | 1047 | 40 (>500Kb only) | 11/15 |
| **Lionel *et al.* ^a^** | Affymetrix Genome-Wide Human SNP Array 6.0 | 2011 | 248 | 2357 | 306 (>20Kb & ≥5 probes) | 170 out of 173 tested cases had inherited CNVs only |
| **Lesch *et al.* ^b^** | Tiling path BAC array | 2011 | 99 | 700 & | 17 | 14 |
| **Stergiakouli *et al.* ^c^** | Illumina Human660W-Quad BeadChip | 2012 | 480 (excluding 319 cases from ) | 5081 | 85 (>500Kb only) | Data not published |
| **Williams *et al.* (2) ^c^** | Affymetrix Genome-Wide Human SNP Array 5.0 | 2012 | 873 (excluding 319 cases from , but including 99 from ) | 2455 | 460 (>100Kb only) | Data not published |
| **Jarick *et al.* ^c^** | Illumina Human660W-Quad BeadChip | 2012 | 489 | 1285 | 51 (>500Kb only) | Data not published |

^a^ In the main text, the cohorts obtained from these publications were combined to form the ADHD-meta cohort.

^b^ This data set was excluded from our analyses because the resolution of the BAC array used is too low to be readily comparable to that obtained in the remaining studies.

^c^ In the main text, the cohorts obtained from these publications were combined to form the ADHD-replication cohort.

**Supplemental Table SII: *De-novo* CNVs present in individuals in the Hyper/SAS cohort**

CNVs highlighted in blue were not included in our analyses because they were <500Kb.

| DECIPHER_ID | *Hyperactivity* | *Short attention span* | Chr | Start | Stop | CNV type | Inheritance status | CNV length |
| --- | --- | --- | --- | --- | --- | --- | --- | --- |
| **128** | Yes | Yes | 8 | 8156705 | 11929473 | LOSS | *de novo* | 3772768 |
| **632** | Yes |  | 17 | 17338154 | 20230301 | LOSS | *de novo* | 2892147 |
| **820** | Yes |  | 8 | 101279091 | 106200542 | LOSS | *de novo* | 4921451 |
| **897** | Yes | Yes | 7 | 72156416 | 73899264 | GAIN | *de novo* | 1742848 |
| **1215** | Yes |  | 2 | 190280619 | 203318141 | LOSS | *de novo* | 13037522 |
| **1351** | Yes |  | 8 | 8009046 | 11730851 | LOSS | *de novo* | 3721805 |
| **1770** | Yes |  | 15 | 32347876 | 33263223 | LOSS | *de novo* | 915347 |
| **1954** |  | Yes | 1 | 66677291 | 72983871 | LOSS | *de novo* | 6306580 |
| **2265** |  | Yes | 6 | 33521322 | 34013145 | LOSS | de novo | 491823 |
| **2370** |  | Yes | 16 | 14993254 | 16189808 | GAIN | *de novo* | 1196554 |
| **2575** | Yes |  | 3 | 105180963 | 118326520 | LOSS | *de novo* | 13145557 |
| **2753** | Yes | Yes | 10 | 130790378 | 133792769 | LOSS | *de novo* | 3002391 |
| **""** | “” | “” | 22 | 44096704 | 48920940 | GAIN | *de novo* | 4824236 |
| **248474** | Yes |  | 15 | 28748072 | 30231488 | LOSS | *de novo* | 1483416 |
| **249134** | Yes |  | 15 | 28719136 | 30298155 | LOSS | *de novo* | 1579019 |
| **250054** |  | Yes | 8 | 79236951 | 82292083 | GAIN | *de novo* | 3055132 |
| **250183** | Yes |  | X | 24736028 | 25452649 | GAIN | *de novo* | 716621 |
| **250380** | Yes |  | 17 | 69779007 | 69974731 | GAIN | de novo | 195724 |
| **250682** |  | Yes | 8 | 42466335 | 54023682 | GAIN | *de novo* | 11557347 |
| **251124** | Yes |  | 17 | 41059585 | 41571264 | LOSS | *de novo* | 511679 |
| **251252** | Yes |  | 17 | 16590367 | 18617705 | GAIN | *de novo* | 2027338 |
| **251421** | Yes |  | 15 | 20090262 | 27091095 | GAIN | *de novo* | 7000833 |
| **251756**^a^ | Yes |  | 2 | 100854899 | 104544452 | LOSS | *de novo* | 3689553 |

^a^ This individual was the only member of the cohort who did not also have *Mental retardation/developmental delay*.

**Supplemental Table SIII: Enrichment analysis of *abnormal learning/memory/conditioning* genes among the 1:1 mouse orthologues of gain-genes present in the DECIPHER Hyper/SAS cohort**

| ***Hyperactivity*-only** | | | ***SAS*-only** | | | ***Hyperactivity* with *SAS*** | | | ***Hyperactivity* and/or *SAS*** | | |
| --- | --- | --- | --- | --- | --- | --- | --- | --- | --- | --- | --- |
| Frac. | FC | *p* | Frac. | FC | *p* | Frac. | FC | *p* | Frac. | FC | *p* |
| 5/22 | 3.3 | 0.015 | 2/13 | 2.2 | 0.2 | 5/20 | 3.6 | 0.01 | 12/55 | 3.2 | 3x10^-4^ |

The first three columns show the results for the sub-cohorts of the Hyper/SAS cohort (wherein individuals are categorised according to their joint *Hyperactivity* and *Short attention span* (*SAS*) status). The last column shows the enrichment analysis for all gain-genes harboured by individuals in the whole Hyper/SAS cohort (reported in the main text), whose determined disruption in mouse yields a phenotype that has been recorded in the MGI (termed “genes annotated with mouse phenotypes”). Key: each of the main columns is divided into three further columns: “Frac.” = the fraction of genes annotated with mouse phenotypes whose orthologues’ disruption yields *abnormal learning/memory/conditioning* in mouse; “FC” = the Fold-change, compared to genes annotated with mouse phenotypes, that this fraction represents; “*p*” = *P-value* obtained using the hypergeometric test.

**Supplemental Table SIV: Co-expressed candidate-genes previously implicated in ASD or SCZ**

Summary of literature review and gene reports acquired from AutDB and SZGene .

|  | **Disease** | |
| --- | --- | --- |
| **Gene** | **Autism Spectrum Disorder** | **Schizophrenia** |
| *CHL1* |  | Association between missense polymorphism in *CHL1* and SCZ in Japanese and Chinese populations |
| *SERPINI1* |  | *SERPINI1* participates in a “network of harboring damaging *de-novo* mutations in Schizophrenia that are co-expressed in fetal Dorsolateral and Ventrolateral Prefrontal Cortex” |
| *APBA2* | Suggested role for *APBA2* in ASD (copy number and sequence variants) . | Suggested role for *APBA2* in SCZ due to *de-novo* duplications |
| *SLC12A6* | *SLC12A6* is located on chromosome 15q14, a region that has shown linkage to ASD, SCZ, Bipolar Disorder, Epilepsy and ADHD. | *SLC12A6* is located on chromosome 15q14, a region that has shown linkage to ASD, SCZ, Bipolar Disorder, Epilepsy and ADHD. |
| *UBE3A* | Studies have found rare variations in the *UBE3A* gene that are associated with ASD. |  |
| *DOC2A* |  | *DOC2A*  is duplicated in patients with SCZ . |
| *MAPK3* | *MAPK3* is located on chromosome 16p11.2, the deletion of which is a risk factor for ASD and causal for ASD in some families . Other studies have identified CNVs in this region in patients with ASD . Overall deletions of 16p11.2 are more penetrant for ASD than the reciprocal duplication . Additionally, rare mutations in the *MAPK3* gene have been identified in ASD . |  |
| *RAI1* | *RAI1* lies in 17p11.2, a region duplicated in a male child with ASD and severe language delay . It was also identified as a susceptibility gene for ASD in a gene network analysis . |  |
| *STX1A* |  | One study found a genetic association between *STX1A* and SCZ , but another found no association . |

**Supplemental Table SV: Mutations in seven of the 14 co-expressed candidate-genes associated with diseases, including neurological or neuropsychiatric syndromes and disorders**

**Data from OMIM , and summarised using DAVID .**

| **Gene** | **OMIM Disease** |
| --- | --- |
| *GTF2IRD1* | Williams-Beuren syndrome |
| *ARX1* | Epilepsy, myoclonic, with mental retardation and spasticity. Epileptic encephalopathy, early infantile. Hydranencephaly, with abnormal genitalia. Infantile spasm syndrome. Lissencephaly, X-linked, with ambiguous genitalia. Mental retardation, X-linked. Otahara syndrome. Partington syndrome. Proud syndrome. |
| *NDN* | Prader-Willi syndrome |
| *RAI1* | Smith-Magenis syndrome |
| *SERPINI1* | Encephalopathy, familial, with neuroserpin inclusion bodies. Many sequence variants affecting diversity of adult human height. |
| *SLC12A6* | Agenesis of corpus callosum with peripheral neuropathy (Andermann syndrome). Agenesis of the corpus callosum with peripheral neuropathy. |
| *UBE3A* | Angelman syndrome. |

**Supplemental Figures**

**Supplemental Figure S1**: **Protocols for determining CNV/ gene overlap**

**
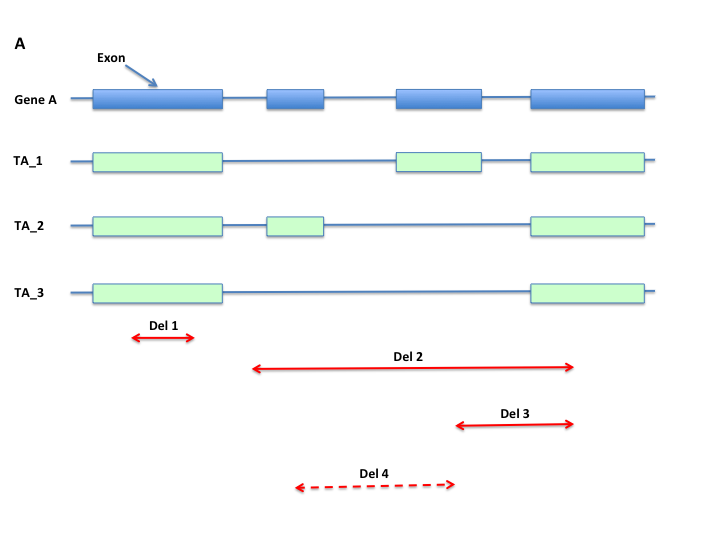
**

**
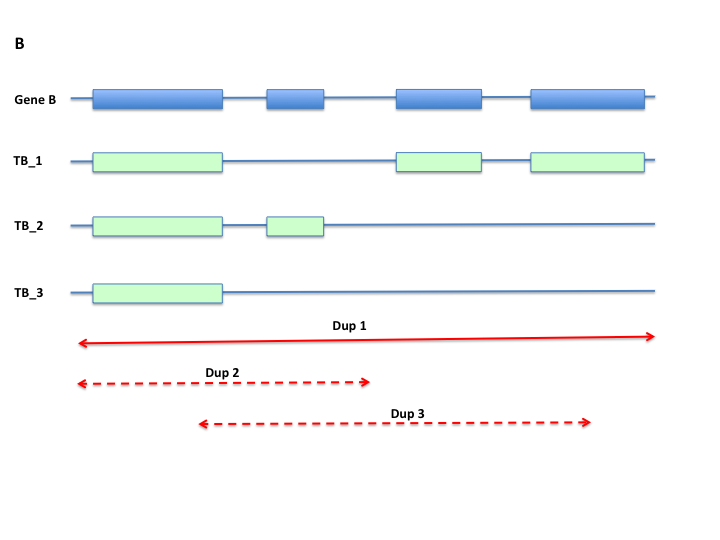
**

In this figure, genes are drawn in blue, gene-transcripts are green, exons are depicted as rectangles, and CNVs are drawn as red, double-headed arrows. (**A**) A gene is labeled as a loss-gene if at least one exon in every transcript is overlapped by the deletion; therefore Del 1, Del 2 and Del 3 would all cause Gene A to be categorised as a loss-gene, but Del 4 (drawn with a dashed line) would not. (**B**) A gene is labeled as a gain-gene when 100% of every transcript is overlapped by a duplication; therefore Dup 1 would cause Gene B to be categorised as a gain-gene, but Dup 2 and Dup 3 (both drawn with dashed lines) would not.

**Supplemental Figure S2: Schematic diagram of the Mammalian Phenotype Ontology (MPO)**

**
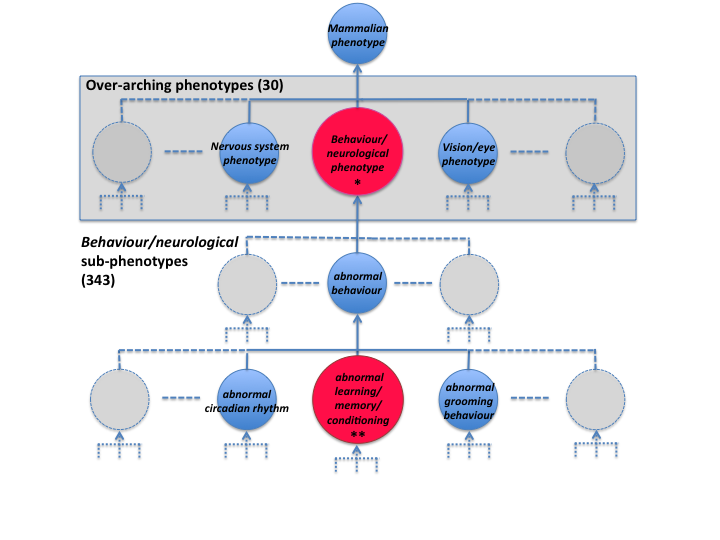
**

There are 30 over-arching terms in the MPO, which we have schematically depicted in the grey box (labeling 3 examples). Beneath the over-arching terms are sub-trees of more specific phenotypes (the blue arrows represent “is-a” relationships; for example, *abnormal behaviour* is-a *Behaviour/neurological phenotype*). Here, we have expanded the sub-tree of *Behaviour/neurological phenotype*, depicting four of the term’s 343 sub-phenotypes. We have highlighted, in red, the phenotypes that were enriched among the 1:1 mouse orthologues of gain-genes from the ADHD-meta cohort (* = significant at 5% (single test); ** = significant at 5% FDR).

**Supplemental Figure S3: Mapping the London Dysmorphology Database terms *Hyperactivity* and *Short attention span* to the corresponding terms in the Human Phenotype Ontology, and hence to ADHD**


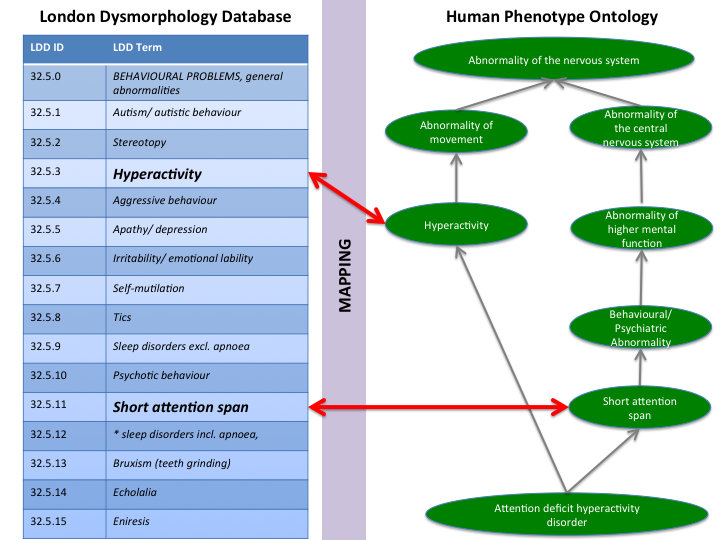


DECIPHER uses terms from the London Dysmorphology Database (LDD, ) to describe the phenotypes present in individuals. The terms available for behavioural problems are shown, with their LDD ID numbers, on the left hand side of the figure. In particular, we see that there is no term specifically for ADHD, however the terms *Hyperactivity* and *Short attention span* are available. One way to ascribe semantic meaning to these LDD terms, and hence relate them to ADHD, is to map them to the Human Phenotype Ontology (HPO, ; mapping downloaded from http://compbio.charite.de/svn/hpo/trunk/src/mappings/LDDB2HPO-v2.csv , via http://human-phenotype-ontology.org), which “aims to provide a standardized vocabulary of phenotypic abnormalities encountered in human disease”. Part of the HPO is shown on the right hand side of the figure, with phenotypic terms drawn in green ellipses, and connected to each other with grey arrows indicating an “is-a” relationship (so, for example, ‘Short attention span’ “is-a” ‘Behavioural/ Psychiatric Abnormality’). We see that there is a 1:1 mapping between the LDD term *Short attention span* and the HPO term of the same name (drawn as a red double arrow); similarly for the LDD term *Hyperactivity*. In addition, in the HPO the term ‘Attention deficit hyperactivity disorder’ directly inherits semantic meaning from only two terms: it “is-a” ‘Hyperactivity’ and “is-a” ‘Short Attention Span’. These facts taken together mean that, in DECIPHER, the presence of ADHD in a patient must be recorded using one or both of the LDD terms *Hyperactivity* and *Short attention span*. Importantly, though, the relationships ‘Attention deficit hyperactivity disorder’ “is-a” ‘Hyperactivity’, and ‘Attention deficit hyperactivity disorder’ “is-a” ‘Short Attention Span’ are not symmetric, so a DECIPHER patient described using the corresponding LDD terms does not necessarily have ADHD.

**Supplemental Figure S4: Distribution of phenotypes among DECIPHER Hyper/SAS cohort**

**A**) Heatmap of phenotypes present in each individual. A yellow square indicates that an individual presented with the corresponding phenotype. Note that the DECIPHER term for ID is *Mental retardation/developmental delay*. **B**) Pie chart of the proportion of individuals in the cohort with *N* phenotypes, when *N* is divided into bins of size 5.


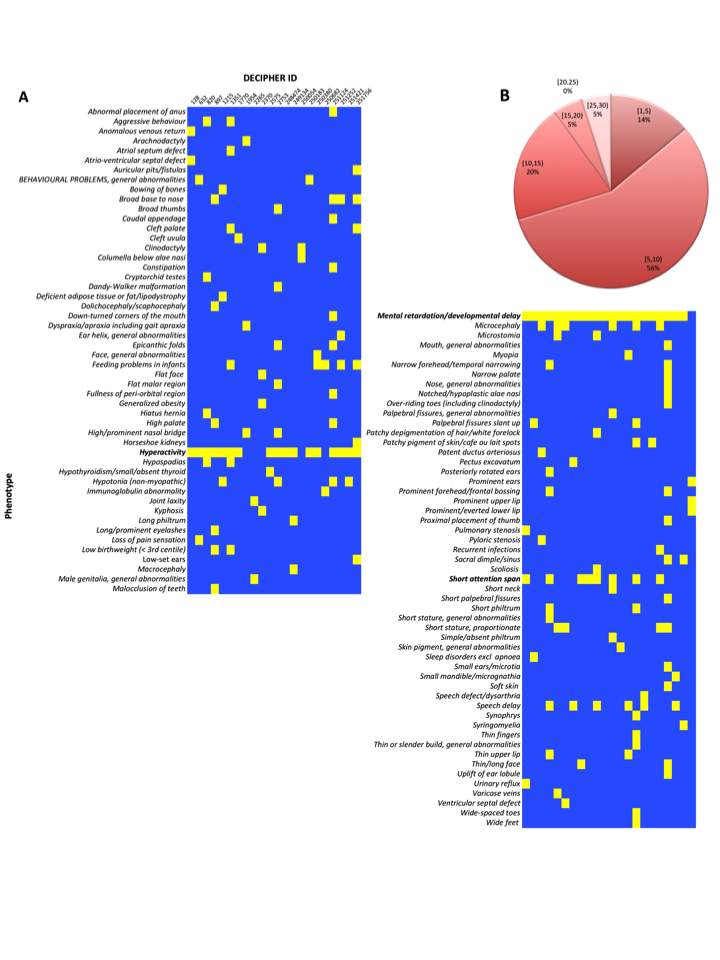


**Supplemental Figure S5: Length distributions of gains >500Kb present in the ADHD-meta and Hyper/SAS cohorts**

Gains >500Kb present in the Hyper/SAS cohort are longer than those arising in the ADHD-meta cohort (*p*=0.003 (Wilcoxon rank sum test)).

**Supplemental Figure S6: Candidate-genes and their connections, within the brain-specific co-expression network, to genes whose orthologues’ disruption yields *hyperactivity* in mouse (termed “genes annotated with *hyperactivity*”).**

The network of genes annotated with *hyperactivity* is depicted in the centre of the figure, highlighted with a light grey oblong, and candidate-genes are shown to the left and right. Six of the candidate-genes’ are also annotated with *hyperactivity* in the mouse, and 5 of these were present in the network (*RYR3* was not); these five genes, depicted at the top of the grey oblong, are included here for a complete picture of the overall network, but were not included in our network analyses. Candidate-genes follow the same key as shown in **Figure 2A** in the main text (Elia-cohort genes = orange circles, Williams-cohort genes = red circles, Lionel-cohort genes = yellow circles, Williams- & Lionel-cohort genes = red circle with yellow border, Hyper/SAS-cohort genes = purple circle, and Williams- & Hyper/SAS-cohort genes = red circle with purple border). Genes annotated with *hyperactivity* (that were not also candidate-genes) are shown as light pink, small circles.

**References**
